# Characteristics and Microstructure of Coatings of Ultradisperse TiB_2_-TiAl Electrodes with Nanosized Additives Deposited on Ti-Gr2 by Non-Contact Electrospark Deposition

**DOI:** 10.3390/ma19030572

**Published:** 2026-02-02

**Authors:** Georgi Kostadinov, Antonio Nikolov, Yavor Sofronov, Todor Penyashki, Valentin Mishev, Boriana Tzaneva, Rayna Dimitrova, Krum Petrov, Radoslav Miltchev, Todor Gavrilov

**Affiliations:** 1 Faculty of Industrial Technology, Technical University of Sofia, 1756 Sofia, Bulgaria; tpeniashki@abv.bg; 2 Department of Material Science and Technology of Materials, Faculty of Industrial Technology, Technical University of Sofia, 1756 Sofia, Bulgaria; anikolov@tu-sofia.bg (A.N.); v_mishev@tu-sofia.bg (V.M.); r_dimitrova@tu-sofia.bg (R.D.); kpetrov@tu-sofia.bg (K.P.); 3 Department of Theory of Mechanisms and Machines, Faculty of Industrial Technology, Technical University of Sofia, 1756 Sofia, Bulgaria; rmiltchev@tu-sofia.bg; 4 Center of Excellence “Mechatronics and Clean Technology”, Campus Studentski Grad, Technical University of Sofia, 1756 Sofia, Bulgaria; 5 Department of Chemistry, Faculty of Electronic Engineering and Technologies, Technical University of Sofia, 1756 Sofia, Bulgaria; borianatz@tu-sofia.bg; 6 Department of Manufacturing Technology and Systems, Faculty of Industrial Technology, Technical University of Sofia, 1756 Sofia, Bulgaria; tgavrilov@tu-sofia.bg

**Keywords:** local electric spark deposition, titanium, roughness, microhardness, phase composition, carbides, borides, nitrides, amorphous, nanostructures

## Abstract

The article considers issues related to improving the surface characteristics of titanium Gr2 using one of the lightest, cheapest and most ecological methods—electrospark deposition with low pulse energy and with ultradisperse electrodes TiB_2_-TiAl with nanosized additives of NbC and ZrO_2_. Using profilometric, metallographic, XRD, SEM and EDS methods, the change in the geometric characteristics, composition, structure, micro and nanohardness of the coatings as a function of the electrical parameters of the ESD regime has been studied. The results show that the use of TiB_2_-TiAl electrodes and low pulse energy allows the formation of dense, continuous and uniform coatings that demonstrate a significant reduction in roughness, inherent irregularities and structural defects of electrospark coatings. Coatings with minimal defects, with crystalline–amorphous structures, with newly formed intermetallic and wear-resistant double and triple phases of the type AlTi_3_, TiAl_3_, TiB, TiN_0.3_, Al_2_O_3_, AlB_2_, TiC_0.3_N_0.7_, Ti_3.2_B_1.6_N_2.4_, Al_2.86_O_3.45_N_0.55_ have been obtained. Possibilities have been found for controlling and obtaining specific values for the roughness and thickness of coatings in the ranges Ra = 1.5–3.2 µm and δ = 8–19.5 µm, respectively. The electrical parameters of the modes ensure the production of coatings with previously known thickness and roughness, with increased microhardness up to 13 GPa, with the maximum possible content of deliberately synthesized high-hard phases and with ultra-fine-grained structures have been defined.

## 1. Introduction

In recent years, due to their unique properties, titanium and its alloys have become increasingly attractive materials and are used intensively in almost all industrial sectors [1,2,3]. However, the relatively low hardness of these alloys, their high chemical activity, their tendency to intense abrasive and adhesive wear and a variable coefficient of friction significantly limit and hinder their use in frictional units and mechanisms of machines [4,5,6] and many other applications. At the present stage, the main strategy for solving this problem is the use of surface modification methods, which are one of the cheapest and most effective ways to create surface layers with high mechanical and tribotechnical characteristics, combined with material economy, product cost reduction and the possibility of their repeated use. Due to the continuously growing interest in the industrial use of titanium alloys, improving their surface properties in order to expand their range of applications has become an important research area worldwide. Researchers are constantly investigating and evaluating methods and coating materials to find the most suitable combinations between the properties of the coated surfaces and the most economical, safe and environmentally friendly methods used. Modern science and technologies provide a variety of such methods, but the most popular of them, such as chemical and physical vapor deposition (CVD and PVD) [7,8,9], ion-plasma nitriding [10], laser [11], thermochemical [12] galvanic, electron beam deposition, microarc oxidation and thermal spraying, cannot always meet all industrial requirements. They are not always applicable due to various limitations, such as energy-intensive and expensive equipment and technologies, high and excessive production costs, environmental pollution, their ability to process only certain materials, complex, unfavorable structural and thermal deformation of the base, as well as the inability to provide the necessary adhesion and thickness, or the necessary physicomechanical, tribological and corrosion properties of the coatings, their inability to produce modification of complex geometric contours, or the need for pre- and post-treatment of the coated surfaces. In many cases, the application of most of the methods mentioned above, which are implemented through complex technologies and require high investments and costs, is not always economically viable and appropriate.

Due to its simplicity and flexibility, electrospark deposition (ESD), with its extremely low cost, easy and universal technology, simple, inexpensive, compact and portable equipment [13,14,15], has a number of advantages, allowing to overcome most of the limitations inherent in the above methods [16,17,18] and motivating its growing popularity, constantly expanding applications and increasingly widespread use for strengthening titanium surfaces. Its main advantages over the above-mentioned methods have been noted in many other works [19,20,21] and are environmental safety, high efficiency at extremely low material and energy costs, universal and maximally simplified technology, one of the strongest bonds of the coating with the substrate compared to other methods, low thermal effect and absence of heating and deformation of the laminated product, possibility of local processing of any surface, even with the most complex shape, ease of performing technological operations, rapid local heating and rapid cooling at a rate (10^5^–10^6^ °C/s) noted in the works [16,17,21,22], which reduces the heat-affected zone and leads to the formation of surface layers with a fine-grained and even amorphous structure, and possibility of applying a wide range of any electrically conductive materials and of changing the characteristics and properties of the coatings in a wide range through the energy parameters of the processing modes. Compared to laser ablation, CVD and PVD processes, thermal methods, ion-plasma nitriding, etc., the ESD method is much more economical and easier to implement and does not require vacuum chambers, complex and expensive equipment, as well as preliminary and subsequent treatments of the deposited surface.

The method is based on the process of electrical erosion in a gas environment and consists of transferring particles of the material from a coating electrode (anode) to the surface of the coated product (cathode) under the action of numerous short-term pulsed electric spark discharges. The layer is formed under conditions of local short-term effect of high temperatures (up to 10,000 °C) and elevated pressures. Under the effect of spark discharges, explosive melting of microscopic particles from the electrode and partial local melting of microscopic areas of the substrate occur. The products eroded from the electrode are transferred at high speed to the surface of the base metal (cathode). When they collide with the surface of the molten cathode micro-area, the transferred particles partially diffuse to a small depth and a mixture of both materials and new compounds and phases are formed, resulting from their chemical interaction and from reactions with elements of the environment. Since the duration of this process is 10^−6^–10^−5^ s, in the time between two consecutive pulses, ultrafast cooling and crystallization of a surface layer occur with fixed high-temperature states, non-equilibrium phases, supercooled modified structure, greatly altered physicochemical properties and increased tribological and corrosion performance.

The material eroded by the electrode is transferred to the substrate in vapor, liquid and solid (softened) phases. The ratio between these phases and the size of the single portions of transferred anodic material depends on the energy of the pulses and the type of anode and cathode materials. ESD on titanium and its alloys is most often carried out at single pulse energies of 0.1–0.6 J, using a wide range of electrode materials—pure metals, alloys and most often hard alloy compositions. Due to the high erosion resistance and high brittleness of the hard alloy electrodes used, a significant part of the transferred material is in the hard (softened) phase and the formed coatings usually have increased roughness, unevenness and structural defects (pores, cracks and cavities), which reduces the effect of their use and limits their applications. According to the cited researchers, the main disadvantages that prevent the even wider spread of ESD for improving the surface properties of titanium and its alloys are the relatively small and uneven thickness of the coatings, the relatively high roughness and unevenness of the surface, the presence of pores and cracks, residual tensile stresses, a decrease in fatigue strength in the surface layer, the difficulty of controlling the homogeneity of the coatings and the uniform distribution of elements, as well as the relatively low productivity and insufficient information regarding the selection of processing modes and electrode materials. Therefore, improving the quality and properties of the coatings and enhancing the ESD effect is associated with the creation and use of more progressive electrode materials and process parameters that ensure the production of coatings with a predominant of liquid phase transfer, the occurrence of exothermic chemical reactions in the interelectrode gap, strong adhesion to the titanium surfaces, higher microhardness and wear resistance combined with low roughness and structural defects.

In recent years, a significant number of studies have been conducted on the development and use of electrode materials that allow the production of coatings with lower roughness and structural defects on titanium surfaces. Cadney et al. [22], Milligan et al. [23], Liu et al. [24], Burkov et al. [25] use aluminum and aluminum alloys to obtain coatings with reduced roughness and structural defects, and coatings made of TiAl alloys [24,25] demonstrate increased corrosion resistance. However, the metals and alloys used by the authors above do not provide sufficiently high hardness and wear resistance. In order to improve the characteristics and wear resistance of titanium surfaces, other researchers use various hard alloy compositions based on TiC and TiN [26,27,28], WC [29,30], TiB_2_ and ZrB_2_ [31,32,33,34] for electrodes. According to these authors, the hardness and wear resistance of titanium surfaces coated with hard alloy compositions exceed those of surfaces coated with metal alloys, but the increased roughness and structural defects of hard alloy coatings reduce the effect of their use and are unfavorable in terms of their corrosion resistance.

As an alternative approach, the production of amorphous and nanostructured surfaces through ESD has emerged. For the formation of amorphous–nanocrystalline and amorphous coatings by selecting appropriate electrodes and electrical parameters of the pulses is reported in Cadney et al. [22], Milligan et al. [23], Xiang Hong et al. [26], E. A. Levashov et al. [28], and Burkov et al. [35]. These and other authors report that the amorphous and nanostructured coatings they have obtained differ from those obtained with conventional electrodes in terms of improved physical and mechanical characteristics, with minor structural defects, increased wear resistance and improved corrosion characteristics. Most of the electrodes used to form amorphous coatings, however, are made of various metal alloys, and despite the improved characteristics of the coatings, their hardness and wear resistance are usually lower than that of coatings obtained from hard alloy compositions. (For example, in the work [31] a 4-fold increase in the wear resistance of AlN–ZrB_2_ coatings is reported; in [32] with an electrode based on TiC-TiB_2_, a pulse voltage of 92 V and a frequency of 1500 Hz the authors report over 13 GPa microhardness of the coatings on Ti6Al4V, while with the metal electrodes used the microhardness of the coatings is most often up to 9–10 GPa). With the development of self-propagating high-temperature synthesis (SHS) technology, a fundamentally new approach to the production of suitable electrodes was discovered. Using SHS technology, authors Levashov, Manakova, Zamulaeva and Kudryashov et al. [36,37,38] created hard alloy electrodes from ultrafine powders, dispersed-reinforced with nanosized additives, and with them managed to achieve both higher microhardness and wear resistance compared to coatings obtained with metals and metal alloys, as well as a reduction in structural defects characteristic of hard alloy coatings. By using these electrodes at relatively low pulse energy and pulse duration up to 60 µs, they produced multifunctional coatings with amorphous–nanocrystalline structures and with higher physical, mechanical and tribotechnical properties, mainly on steel surfaces. The most promising of these electrodes are the TiB_2_-TiAl nano electrodes [37,39,40], which are used to create coatings with reduced roughness and surface defects, increasing the service life of coated steel tools and parts by 2–5 times. These results outline a promising direction for improving the structure and properties of electrospark coatings. However, the study of the literature data showed that the use of SHS electrode materials for strengthening titanium surfaces has been poorly studied and there is still no comprehensive data on the specifics of formation and characteristics of coatings from these promising electrodes on titanium surfaces.

In this context, the aim of this work is to investigate the possibility of using the ESD method on titanium with a TiB_2_-TiAl nano electrode created by SHS [37,38,39,40], by studying the relationships between the parameters of pulse discharges and the topography, composition and structure of the coated titanium surfaces and to define appropriate energy conditions for creating coatings with reduced roughness and structural defects and parallel local synthesis of new wear-resistant phases and ultra-fine-grained (UFG) structures with increased hardness.

The tribological and corrosion behavior of these coatings, as well as the influence of the energy parameters for their deposition on their tribological and corrosion characteristics will be examined in our further research and will be published in our next work, which appears as a continuation and development of the research and results presented here.

## 2. Materials and Methods

### 2.1. Electrode Selection

An ultradisperse electrode, developed by self-propagating high temperature synthesis SHS with the composition TiB_2_-TiAl nano (which will be called TiB_2_-TiAl for short) (74%Ti + 12%B + 14%Al) dispersely reinforced with 7% nanosized ZrO_2_ and additives of NbC particles, created by the authors [38,39,40], was used to form coatings with amorphous and nanostructured phases and to increase the microhardness and wear resistance of the coated surfaces.

Cylindrical electrodes with a diameter of 1.5 mm and a length of 40 mm were used, which were cut by electrical discharge machining (EDM) from samples with dimensions of 6 × 4 × 40 mm.

Titanium borides have a high hardness (≈34 GPa) and high chemical resistance, which creates prerequisites for high tribological and anti-corrosion characteristics. In the works [32,33,34] the high adhesion, hardness and wear resistance of hardalloy coatings based on TiB and TiB_2_ were reported. The effectiveness of TiB_2_ as a coating material, used in other methods of surface modification, has been demonstrated in many other works [41,42]—for laser cladding coatings, [43,44,45]—of plasma, flame and arc—sprayed coatings on steels.

However, the use of titanium borides is limited due to their high brittleness and resistance to electroerosion. This problem was solved adding a metallic binder TiAl, which leads to wetting of the wear-resistant solid phase. The intermetallic binary system Ti-Al has a low density, high strength and high temperature resistance and is designed to provide both high adhesion to the substrate and the formation of wear-resistant aluminum borides and oxides [22,25,46]. Nanosized additives have been introduced to act as modifiers in the coating formation process to improve the deposition process and coating quality, as well as to form a favorable combination of high hardness, oxidation resistance and low friction coefficient [38,39,40].

### 2.2. Substrate

Technical titanium Ti-Gr2 (AISI UNS R R56200 and R50400) was used as the substrate. In the initial studies for the initial selection of the parameters of the regimes, preliminary depositions were performed on sheet material with a thickness of 2 mm, and for the subsequent studies, plates with dimensions of 12 mm × 12 mm × 5 mm obtained by electrical discharge cutting (EDM) and subsequent grinding to a roughness Ra ≈ 2–2.5 μm were used.

### 2.3. Research Apparatus

**Figure 1:**
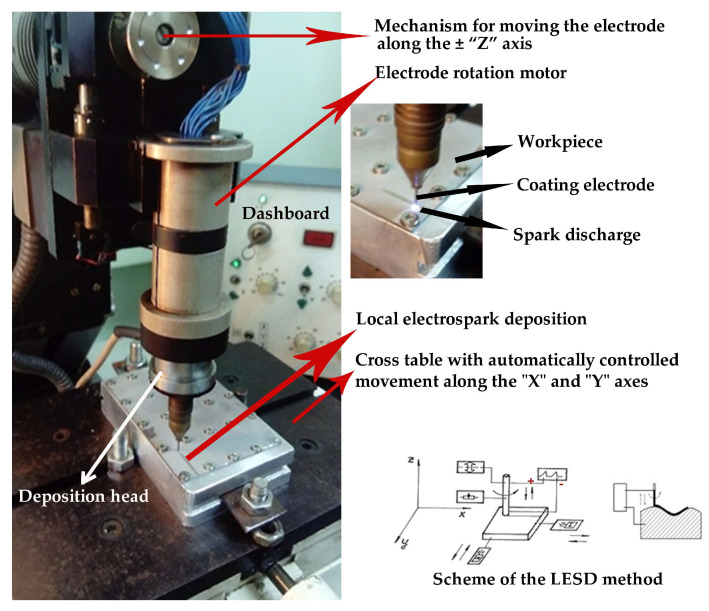
Machine for LESD type “ELFA”.

In order to reduce the size of erosion craters on the substrate surface and obtain a smoother and more uniform layered surface with minimized surface defects, in this work a variation in the classical electrospark deposition contactless local electrospark deposition (LESD) [47] with low pulse energy—0.005 to 0.045 J—was used, since this method produces dense and uniform coatings with low roughness and provides an acceptable combination of characteristics and properties suitable for simultaneously increasing wear resistance and corrosion resistance. The coatings were applied using a mechanized machine type “ELFA” (Sofia, Bulgaria)—Figure 1—with a rotating electrode, automatic maintenance of the discharge distance and controlled deposition speed of 0.6 mm/s. During coating, the workpiece moves at a controlled speed along the X and Y axes. An automatic electrode gap regulator maintains the required gap for the realization of spark discharges and ensures process stability. The gap between the electrodes can be maintained along all three axes, but in this case the coating is applied with the tip of the electrode. The process control was implemented by changing the electrical parameters of the mode.

**Figure 2:**
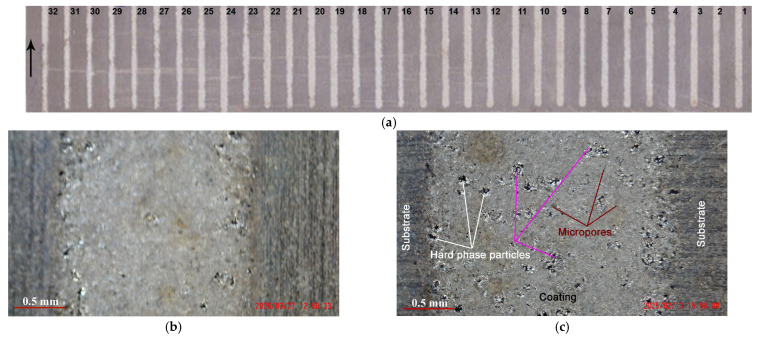
General view of LESD coatings deposited with TiB_2_-TiAl electrode on Ti-Gr2 substrate. (**a**) General view of LESD coatings in modes with pulse energy 0.005–0.045 J. (**b**) Surface of LESD coating in mode 3, Table 1—E ≈ 0.013 J. (**c**) General appearance of LESD coating in mode 1, Table 1—E = 0.025 J.

The studies were conducted with pre-optimized process parameters and pulse energy. Based on a comparison of the roughness, thickness and surface relief of the coatings deposited in 32 modes (Figure 2a) with different values of the pulse parameters in the range: (current = 11.2–24 A, capacitance C = 0.22–5 μF, pulse duration Ti = 5–20 μs and pulse frequency f = 5–20 kHz at pulse duty cycle 0.1. In the study, 5 modes were selected in the following range: lower limit—pulse energy (pulse parameters), in which uniform coatings with a fine structure and a minimum thickness of ≈10 μm were obtained (Figure 2b); and upper limit—the maximum possible pulse energy (pulse parameters), which produces relatively uniform coatings with minimal irregularities and structural defects and with low transfer from the solid (softened) phase.

The pulse parameter values of the selected modes are shown in Table 1. The selected mode parameters are consistent with both our previous studies [48,49,50,51] and the data in the works [32,33,34,37,38,39,40] that use ESD technique with reduced pulse energy to prepare TiB–TiB_2_ coatings with uniform fine structure and higher heat resistance and wear resistance compared to coatings made of hard alloy electrodes based on titanium carbide. The coatings were deposited with three consecutive electrode passes. It is widely known, that with each subsequent electrode pass the rate (gradient) of coating thickness growth decreases, but the thickness continues to grow until reaching the so-called “threshold of brittle failure of the coating” [14,17,18], at which the coating thickness stops growing and shows a tendency to decrease. Preliminary studies showed that the coating thickness increases significantly until the third electrode pass, after which the rate of growth decreases with each subsequent pass, and the thickness increases insignificantly (barely by a few micrometers). Therefore, in this study it was assumed the coatings to be deposited with three consecutive electrode passes, i.e., with a diameter of 1.5 mm and a deposition speed of 0.6 mm/s, the productivity for the three passes is ≈5.5 min/cm^2^. The selected number of passes is also consistent with the time recommended by the electrode manufacturers [38,39,40] until the brittle fracture threshold of 6 min/cm^2^ is reached.

### 2.4. Measurement Methodology

Surface relief, microhardness, thickness, composition, structure and wear resistance were used as the main indicators to evaluate the properties of LESD-modified titanium surfaces.

- The roughness parameters of the coatings (average roughness—Ra; root mean square rough-ness Rq; maximum profile height—Rt; the average value of the 5 highest protrusions and 5 deepest depressions of the profile within the basic length—Rz) were measured with the profilometer “AR-132B” (Shenzhen Graigar Technology Co., Ltd., Shenzhen, China) in two mutually perpendicular directions in five sections. The number of parallel measurements is 5; the measurement length is 2.5 mm. The arithmetic average values, standard deviation and confidence interval were determined. Significantly different values were rejected using the Grubbs method.
- The thickness δ was measured with a dial indicator with an accuracy of 0.001 mm. The results are the arithmetic mean of 5 parallel measurements.
- Vickers microhardness (HV) was measured from above (on the top of the coating) after smoothing the surface irregularities. The hardness tester “Zwick 4350” (Zwick Roell, GmbH & Co., KG, Ulm, Germany) equipped with a Vickers diamond prism indenter at a load of 0.2 N for a time of 10 s was used. The number of parallel measurements was 10. In order to eliminate the influence of the substrate, the measured hardness was calculated according to the method presented in [52].
- To obtain a more accurate assessment of the properties of the coatings, the universal hardness HU was used, which characterizes both the elastic and plastic properties of the material. The measurements were carried out with a computer-controlled FISCHERSCOPE^®^ H100 (Helmut Fischer GmbH, Sindelfingen, Germany) nanotester at a load of 300 mN with a Vickers diamond indenter and a penetration depth of 0.8–1.6 μm. The average values of the 10 measurements performed on each Sample were taken.
- Microstructural, topographic and morphological analyses and the distribution of elements of the coatings were performed with the metallographic optical microscope “Neophot 22” (Carl-Zeiss, Jena, Germany) and the scanning electron microscope (SEM-EDS) “EVO MA 10 Carl Zeiss” with a built-in X-ray energy-dispersive microanalyzer EDX system from “Bruker” (Bruker AXS, Karlsruhe, Germany).
- The phase composition was investigated with a Bruker D8 Advance X-ray diffractometer (Bruker AXS, Karlsruhe, Germany) in “Cu Kά” radiation. The X-ray spectrum was recorded in the angular range from 5.3 to 80° 2θ with a step of 0.03° 2θ and a counting time of 52.5 s/step. Qualitative phase analysis was performed using the PDF-2(2009) database of the International Center for Diffraction Data (ICDD).

## 3. Results and Discussion

### 3.1. Coating Characterization—Roughness, Thickness δ, and Structure of Coatings

**Figure 3:**
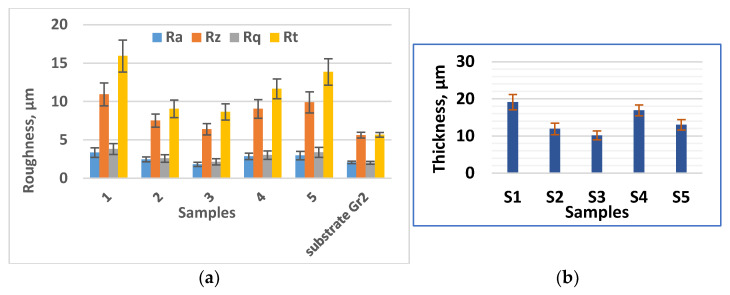
Roughness and thickness of TiB_2_-TiAl^nano^ coatings on Ti Gr2 vs. the pulse energy (substrate R_a_ ≈ 2.5 μm). (**a**) Roughness parameters of LESD coatings. (**b**) Thickness of LESD coatings.

As can be seen from the profilometric results, the values of the roughness parameters of the coated Samples are higher than those of the substrate and for the studied energy range vary within the limits of Ra = 1.8 to 3.2 μm, and the thickness δ—from 9 to 19.5 μm. Within the range specified above, the roughness and thickness of the obtained coatings can be adjusted by the LESD modes. With the increase in energy (current I, capacitance C and pulse duration Ti) the roughness parameters and coating thickness also increase, with the highest values recorded for Sample 1, i.e., at the highest used values of C, Ti and I (Figure 3a,b). The lowest roughness and thickness are recorded for Sample 3, where the pulse energy is also the lowest. The strongest influence on the change in roughness and thickness of the coatings is exerted by the capacitance C and the duration of the pulses Ti, followed by the current I.

**Figure 4:**
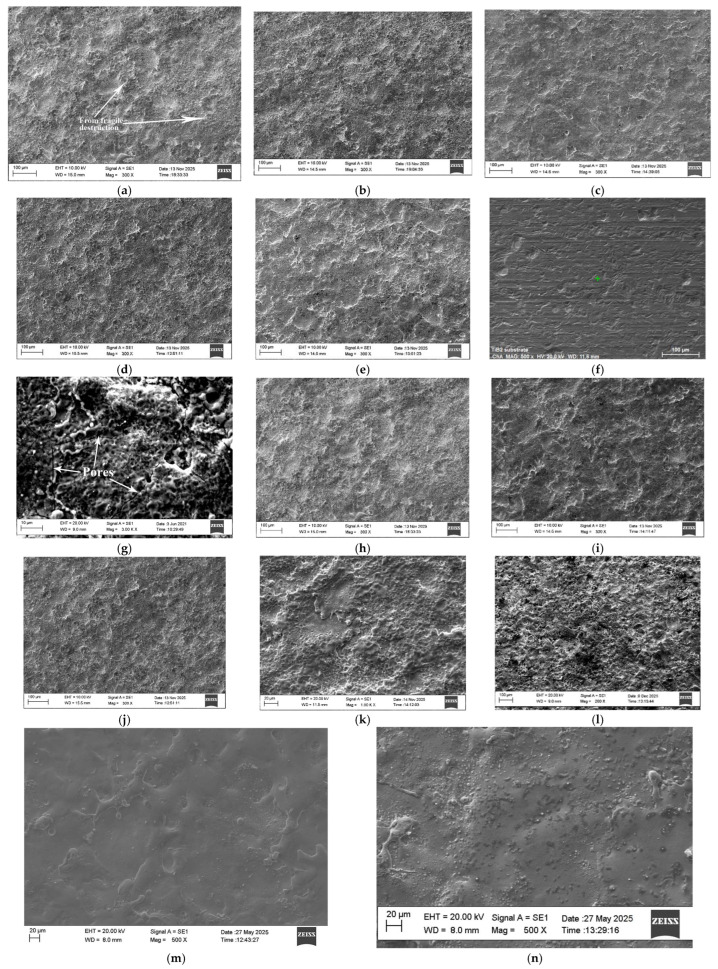
SEM images of surface view on top of TiB_2_-TiAl nano coatings. (**a**) Sample 1—I = 16 A, C = 4.4 µF, Ti = 12 µs. (**b**) Sample 2—I = 11.2 A, C = 0.5 µF, Ti = 12 µs. (**c**) Sample 3—I = 12.8 A, C = 0.5 µF, Ti = 8 µs. (**d**) Sample 4—I = 16 A, C = 2.2 µF, Ti = 12 µs. (**e**) Sample 5—I = 12.8 A, C = 4.4 µF, Ti = 8 µs. (**f**) Ti-GR2—substrate. (**g**) Sample 1—I = 16 A, C = 4.4 µF, Ti = 12 µs. (**h**) Sample 5—I = 12.8 A, C = 4.4 µF, Ti = 8 µs. (**i**) Sample 4—I = 16 A, C = 2.2 µF, Ti = 12 µs. (**j**) Sample 3—I = 12.8 A, C = 0.5 µF, Ti = 8 µs. (**k**) Sample 1—I = 16 A, C = 4.4 µF, Ti = 12 µs. (**l**) Sample 1—I = 16 A, C = 4.4 µF, Ti = 12 µs. (**m**) Sample 3—I = 12.8 A, C = 0.5 µF, Ti = 8 µs. (**n**) Sample 4—I = 16 A, C = 2.2 µF, Ti = 12 µs.

**Figure 5:**
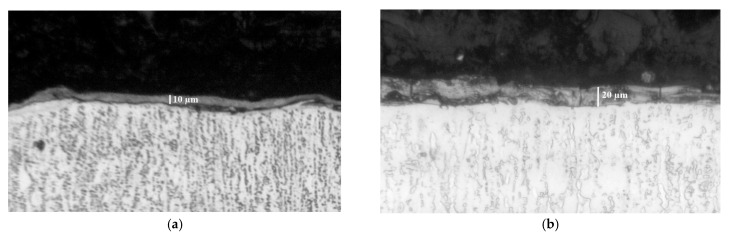
Cross-section microphotographs of microstructure of coatings from TiB_2_-TiAl electrodes. (**a**) Sample 3—pulse parameters I = 12.8 A, C = 0.5 µF, Ti = 8 µs. (**b**) Sample 1 pulse parameters I = 16 A, C = 4.4 µF, Ti = 12 µs.

Figure 4 presents typical SEM images, at different magnifications, of the general view of the surface topography of the coated surfaces in the experimental modes, and Figure 5 shows the cross-sections of Samples 1 and 4.

It is evident that the coatings form a structure with a specific relief on the titanium surface, formed mainly with the participation of the liquid phase. On the samples, convex areas (accumulations of incompletely melted electrode particles), glass-like smooth areas (Figure 4h,i,k,m,n) and those formed by small particles with slight irregularities are distinguishable. It can be seen that the most uniform surface is from the coatings obtained at energy up to 0.015 J. In the coatings applied with the lowest pulse energy (lowest values of I, C, Ti—Samples 2 and 3) the irregularities are smaller, the surface is smoother and more homogeneous, the structural elements that make them up are smaller in size, and microcracks are almost not detected. With increasing pulse energy (in the e-d-a direction—Figure 4—Samples 5, 4, 1—Table 1) a gradual increase in the roughness and irregularities of the coatings is observed. At the highest used energy (I = 16 A, C = 4.4 µF and Ti = 12 µs—Sample 1—Figure 4a,k,l) the highest irregularities are observed, as well as single separate accumulations caused by the transfer of incompletely melted electrode particles, cavities and micropores clearly distinguishable in Figure 4g,k,l. At the next highest pulse energy—Sample 4—the surface of the coating is more homogeneous and smoother, and micropores and irregularities are almost not observed (Figure 4d,i). The inhomogeneous surface of the coatings is obvious. Within the same coating (Figure 4a,k,l) different areas with a predominant light or dark color are observed, which is expected considering the multicomponent composition of the electrode and the impossibility of its complete homogenization. The study also identified areas of defects such as pores and unmelted particles in the structure of the coatings, which are visible in Figure 4g,l,k and Figure 5. Unmelted particles can adversely affect the adhesion of the coating and or serve as starting points for the development of microcracks and other defects. The relief of the coated surfaces is consistent with the data from the measurements of the roughness parameters and the thickness of the coatings—Figure 3—from which it is established that the highest values of the roughness parameters, as well as the thickness of the coatings up to 19.5 μm, were measured for Sample 1—Figure 3a and Figure 4a,k,l. In Figure 5, in cross-sections, the differences in the morphology of the coatings applied at the minimum and maximum pulse energy used can be seen.

From the microsection photographs it is seen that the layer differs from the substrate material in structure and grain size. Micro irregularities are observed in different places on the surface of the coatings.

The interaction between the electrode and the substrate melt leads to the formation of a transition interface layer tightly bonded to the substrate (in the photos—a thin white area immediately under the dark coating), formed as a result of the diffusion penetration of the molten particles into the surface of the titanium substrate, their mixing with the titanium from the substrate and their rapid cooling, which suggest a strong metallurgical bond. Since the amount of material transferred by the electrode and the size of the molten cathode spot increase with increasing pulse energy, the width of the bright interface zone also increases with increasing pulse energy and reaches 4–5 µm for Sample 1 (Figure 5b), while for Sample 3 it is of the order of 1–2 µm. Moreover, no elongated morphology of the substrate is observed in this zone, which could be attributed to the recrystallization caused by the ultrafast cooling of the molten cathode spot and the formation of a fine-grained structure. In depth, this structure passes into the structure of the base material. The wavy surface and the elongated morphology of the substrate are from the original substrate surface. The Ti-Gr2 plates are cut from a square profile measuring 12 mm × 12 mm, obtained by drawing through a die. The elongated morphology of the substrate is most likely a result of drawing through the nozzle. The structure is similar to the other photos taken. While the coatings deposited at the minimum pulse energy are dense and uniform, those deposited at the maximum energy are almost twice as thick (Figure 5b) but also have more irregularities and the presence of rare cracks and cavities. Almost all researchers of the process explain the appearance of pores and irregularities as a result of surface tensile stresses, with the mechanical properties of the electrode material and the excessively high pulse energy used. With an increase in the pulse energy above certain limits, the proportion of the transferred material from brittle fracture increases sharply, which worsens the quality, uniformity, roughness and structural defects of the resulting coatings. In addition, the increased pulse energy leads to other negative effects, such as overheating of the electrode, the appearance of a thermally affected sublayer, and the appearance of cracks and defects in the coatings, from which it follows that in order to avoid the above-mentioned defects, the energy must be increased to certain limits, which, as shown by the data in the literature and those from our previous studies, are different and specific for each specific electrode material. Therefore, in order to obtain a dense layer without microcracks, it is necessary to maintain the level of pulse energy and the most influential and in this case pulse parameter—the capacitance C—below certain values. Based on the obtained data (Figure 2, Figure 3, Figure 4 and Figure 5) and the results of our previous studies [48,49,50,51] in the case of ESD with the TiB_2_-TiAl electrode, this level can be determined as C ≤ 2.2 µF. The SEM images shown in Figure 4 confirm that by ESD with the electrodes used and low pulse energy E = 0.013–0.025 J (Samples 2–4, Table 1), the presence of microcracks and surface roughness was significantly reduced compared to those obtained in the works [31,32,34] in the case of ESD with a vibrating electrode. The TiB_2_-TiAl coatings obtained in this work have a smoother and more uniform surface than those obtained with classical hard alloy electrodes and indicate that by LESD it is possible to obtain smooth coatings almost without structural defects but with a smaller thickness.

### 3.2. Phase Composition and Microhardness of the Coatings

**Figure 6:**
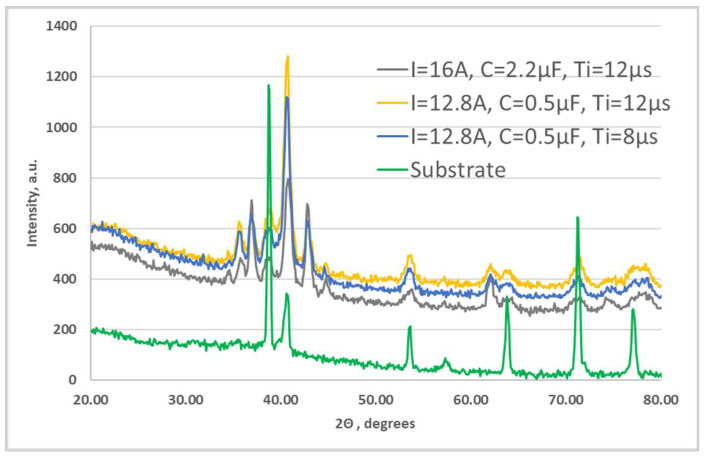
XRD diffraction patterns of coatings from TiB_2_-TiAl electrode at different pulse energies.

Figure 6 shows the diffractograms of LESD Samples 2, 3 and 4, and Table 2 gives the phase composition of the coatings and the values of the angle 2θ at which the characteristic peaks of the corresponding phases are registered. The X-ray diffraction patterns of the coatings applied in the other selected modes—Table 1—are similar.

Since each of the registered characteristic peaks at a specific angle 2θ corresponds to several different phases, the table lists all available phases registered in the presence of at least three diffraction peaks.

As can be seen from Figure 6, the X-ray diffraction patterns of the coatings are similar and their phase composition is close. The main differences are in the intensity and width of the characteristic peaks of the phases. In all coatings, the main phases of the electrode TiB_2_, TiAl are observed, but new compounds are also registered that are not present in the electrode and substrate such as Ti_3.2_B_1.6_N_2.4_, TiB, TiN, TiN_0.3_, Ti (CN), TiC_1−x_. Moreover, the amount of TiB_2_ is much less than in the hard alloy electrode material, which indicates its dissociation (or transformation). Small amounts of Al_2_O_3_, TiC_0.3_N_0.7_, TiC_1−x_, Ti_6_O, Ti_2_O and traces of Ti_3_O, AlN, AlB, BN, Al_2.86_O_3.45_N_0.55_ are also observed. Each new phase has its own unique properties that directly affect the microstructure and properties of the coating. XRD results show that in the LESD process TiB_2_ is partially decomposed to TiB and Ti, and the formed titanium nitrides, carbides, oxides and sets of phases with a general composition Ti (N,C) are due to the reactions of both titanium from the electrode and that from the base with nitrogen, oxygen and carbon from the surrounding air. The short contact time of the particles with oxygen from the environment creates favorable conditions for minimizing oxides in the coating. Increasing the pulse energy leads to an increase in the amount of transferred material, an increase in the degree of partial decomposition of TiB_2_ and, accordingly, an increase in the amount of high-hard and newly formed phases and the degree of alloying of the layer, as well as that of titanium oxides, the amount of which in Samples 1 and 5 reaches about 5–5.8%. The structural maxima also broaden and shift relative to those of the substrate, which is most pronounced in the coatings in Sample 4—Table 1—(I = 16 A, C = 2.2 µF, Ti = 12 µs, f = 8.33 kHz) and is an indicator not only of the presence of internal stresses and solid solutions, but also of the refinement of the structure, which even suggests reaching an amorphous state. The high cooling rate, which according to the authors [15,17,22] reaches 10^5^–10^6^ °C/s, undoubtedly allows the formation of amorphous phases on the substrate surface. Due to the lower pulse energy used in this work and the much shorter pulse duration of 8 and 12 µs, the transferred portions of molten electrode material are much smaller, and the sizes of the molten cathode spot are also much smaller, which creates prerequisites for a higher cooling rate and with a high degree of probability it can be expected that coatings with a fine structure and with nanocrystalline and more amorphous structures will be formed on the cathode than when using conventional hard alloy electrodes and the usual higher pulse energy and pulse duration used in the ESD process. The presence of “glass-like” zones (Figure 4h,k,m,n) also supports the assumption of some partial “amorphization” of the coating and gives reason to assume that with the studied TiB_2_-TiAl electrodes, zones with a structure close to the amorphous are obtained in the coatings by LESD, which we can call pseudo-amorphous structures. At higher pulse energy—Samples 1 and 5 (C = 4.4 µF)—the size and volume of the cathode spot increase, the cooling rate of the melt decreases and, accordingly, the amount of glassy pseudo-amorphous zones also decreases. However, the presence of peaks in the extended angular zones also suggests the presence of crystals. Table 3 shows the range of variation in the crystallite sizes of the main registered phases in the five modes used for LESD. As can be seen from the table, the crystallite sizes calculated by the Scherer formula for the different phases and modes are in the range of 6–87 nm, which indicates the presence of nanosized crystal structures in the layer, and gives reason to assume that with the studied TiB_2_-TiAl electrodes, metal matrix coatings with partially included pseudo-amorphous–nanocrystalline structures and embedded high-hard wear-resistant particles were formed through LESD.

As can be seen from Figure 6, for the coatings obtained with a pulse energy of 0.025 J (Sample 4—I = 16 A, C = 2.2 µF, Ti = 12 µs), the broadening of the characteristic peaks at angles 2θ ≈ 35–40°, 43–46°, 53–56°,61–65°, 70–75° is the largest, therefore it can be assumed and to expected that this coating has more pseudo-amorphous phases than the other coatings. In the coatings obtained with this mode, the number and amount of high-hard phases and intermetallic compounds, the degree of amorphization and the surface hardness are higher than those of the coatings obtained with the other modes. Apparently, the pulse energy of 0.01–0.025 J at a capacity of C ≤ 2.2 µF and Ti = 8 and 12 µs creates smaller sizes of the spot of the molten cathode and, consequently, a sufficiently high cooling rate of the mixed melt, obtaining coatings with an increased amount of amorphous phases with reduced roughness, increased density and uniformity. Due to the reduced amount of structural defects, such as voids, asperities and micropores, the TiB_2_-TiAl coatings deposited at the used modes suggest a higher level of mechanical properties, exceeding the level of properties achieved with currently used electrodes. Because of its low cost and easy technology, the ESD process emerges as a technically and economically feasible alternative for the creation of amorphous and nanosized surfaces, but for this purpose, in-depth research is needed to study both the conditions for creation and the properties of these surfaces, which are still insufficiently studied in the literature.

**Figure 7:**
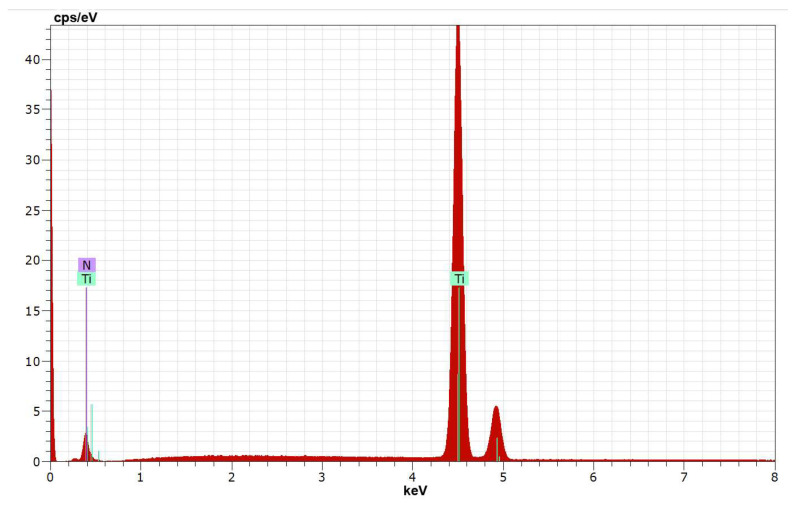
The EDX spectrum of the Ti-Gr 2 substrate.

**Figure 8:**
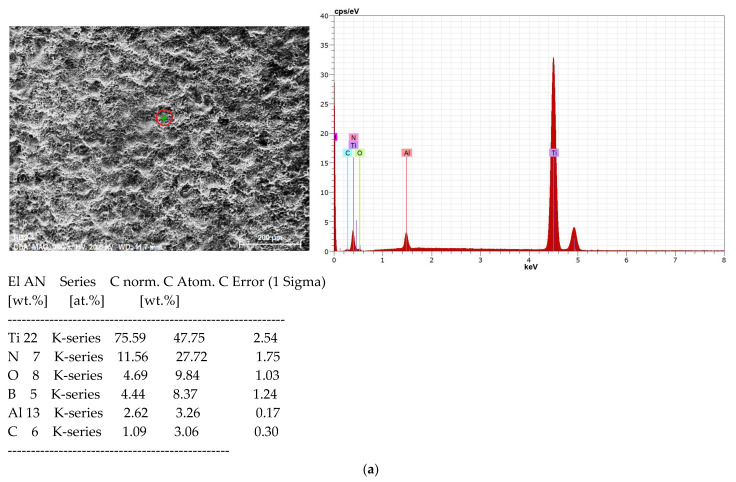

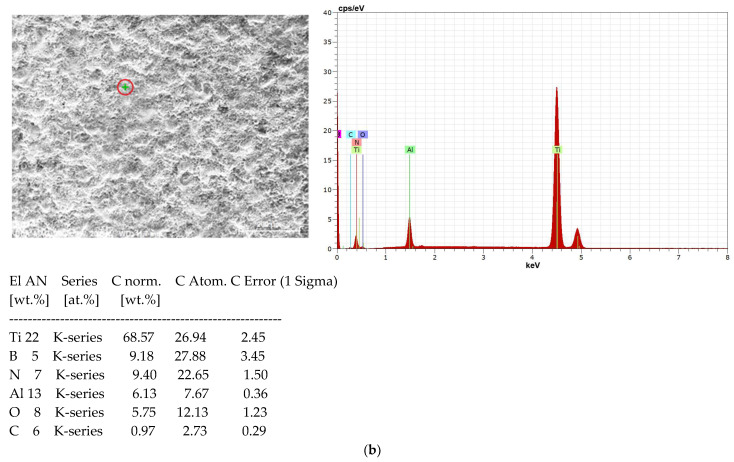
EDX spectrum of coating TiB_2_-TiAl—Sample 4. (**a**) Distribution of elements in Zone 1. (**b**) Distribution of elements in Zone 2.

Figure 7 presents the elemental composition of the substrate, and Figure 8 shows the distribution of elements on Sample 4 with high pulse energy—Table 1—in two different zones, marked on the SEM images with a green cross. As can be seen from the SEM images, Zone 1 (Figure 8a) is located in a low part (a recess) of the coating, and Zone 2 (Figure 8b) is localized on the surface of the accumulation caused by transferred incompletely melted electrode material. In both zones of the coating, the presence of the main elements of the electrode and the substrate, as well as the elements of the surrounding air environment, N, O and C, was recorded. In all coatings, Ti from the substrate prevails.

**Figure 9:**
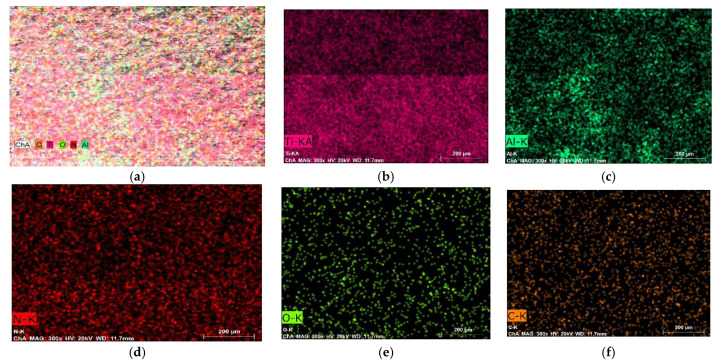
Map data of coating TiB_2_-TiAl—Sample 4. (**a**) General view. (**b**) “Ti” distribution. (**c**) “Al” distribution. (**d**) “N” distribution. (**e**) “O” distribution. (**f**) “C” distribution.

The comparison of the distribution in the two zones shows more than twice the amount of Al and B in Zone 2 and a lower amount of Ti and N, which indicates a higher presence of electrode material. The carbon and oxygen content also differ, but the differences are relatively small. The higher amount of O_2_ in Zone 2 indicates that in the transfer process the electrode material is oxidized to a greater extent than the cathode. The observed differences in the content of the elements prevent an accurate assessment of the distribution, as well as an accurate assessment of the differences in the content of individual elements in the composition of the samples coated in the different studied modes. The clearly expressed heterogeneity of the coating is confirmed by the distribution map—Figure 9. With such a variation in the composition, it can be expected that the mechanical properties will also vary. The presence of oxygen, nitrogen, carbon and boron in the composition of the samples confirms the data from the X-ray structural analysis and testifies to the formation of new phases in the LESD process—such as titanium and aluminum nitrides, carbides and oxides. Moreover, the distribution of oxygen repeats the distribution of both titanium and aluminum, which confirms the presence of titanium and aluminum oxides. However, the distribution of N and C repeats only that of Ti, which suggests the presence of titanium carbides, nitrides and carbonitrides.

The EDX mapping data of the other studied samples are similar, with the difference that at higher energy parameters of the pulses the content of Al, O, N, C is also relatively higher, which corresponds to the larger portions of anodic material transferred to the cathode, but the heterogeneity of the coatings does not allow for an exact comparison.

**Figure 10:**
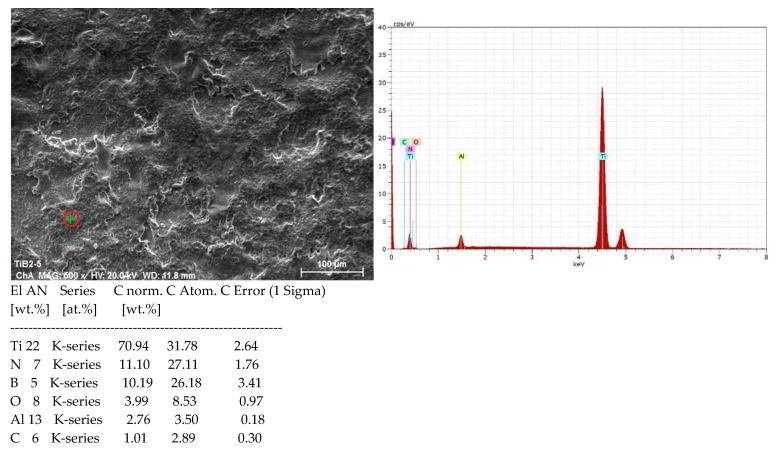
EDX spectrum of TiB_2_-TiAl coating—Sample 5—distribution of elements.

**Figure 11:**
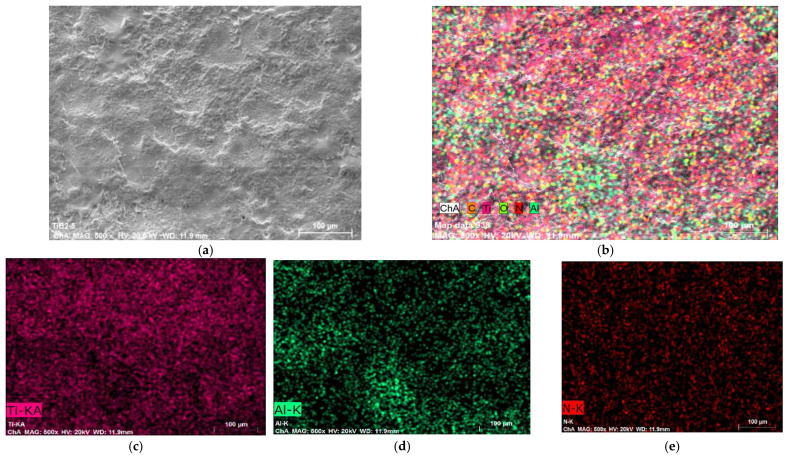

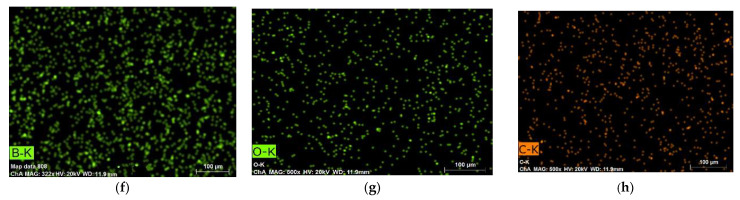
Map data in Sample 5EDX of TiB_2_-TiAl coating—Sample 5. (**a**) SEM image. (**b**) General view. (**c**) “Ti” distribution. (**d**) “Al” distribution. (**e**) “N” distribution. (**f**) “B” distribution. (**g**) “O” distribution. (**h**) “C” distribution.

Figure 10 and Figure 11 show the spectrum and the map of the distribution of elements of Sample 5 with a coating applied in mode 5—Table 1. As can be seen from Figure 10—EDX—the data for Sample 5 obtained at higher capacitance values, but lower values of the flux I and the pulse duration Ti, are similar to those in Figure 8, and the content of N,O,C,Al—Figure 10—in areas with a structure close to that in Figure 8b, is also close. Comparison of the EDX analysis data shows that with increasing pulse energy, the amount of elements from the electrode material also increases at the expense of decreasing the amount of Ti (Figure 8 and Figure 10). As can be expected, the maximum amount of elements from the electrode is recorded in the coatings at the samples with the highest pulse energy 1, 4, and 5—Table 1. Oxygen is detected in the coatings, which is an indicator of oxidation in the process of spark discharges.

**Figure 12:**
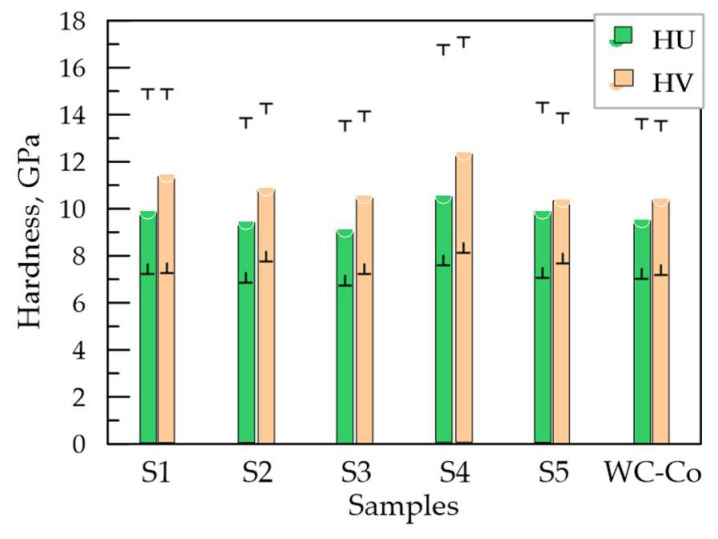
Average, minimum and maximum values of microhardness HV and Universal Hardness HU of LESD coatings from TiB_2_-TiAl electrode under different regimes.

Figure 12 shows the average values, as well as the minimum and maximum measured values of the microhardness HV and the Universal nanohardness HU of the studied samples. For comparison, the values of the hardness and thickness of the coatings from the classical WC-Co8 electrodes, deposited in Sample 4, are also given—Table 1.

From Figure 12 it can be seen that after LESD the average microhardness HV of the coatings significantly increases compared to that of the base 3.22 GPa and for the different modes is in the range 10.6–13 GPa, and the universal hardness HU, respectively, in the range 9.2–11.6 GPa, exceeding that of the titanium substrate by a factor of 3 to 4.5. The microhardness obtained in the individual measurements varies in a very wide range (the lowest measured value is 7 GPa, and the highest 17.7 GPa), but the average values for the studied coatings are relatively close and are slightly higher, comparable to the hardnesses obtained for the coatings from the WC-Co8 electrode in Sample 4. The obtained microhardness values are also comparable with the data presented by Mesut Gökçe et al. [32], who obtained coatings with hardness HV 8.5–13 GPa on Ti_6_Al_4_V (Gr 5) using a TiC + TiB_2_ + Co composite electrode. Kovacik et al. [34] also deposited TiB_2_ on Ti_6_Al_4_V, reporting a hardness of the coatings HV = 10 GPa. The large differences in the individual measured values of micro and nanohardness within the same coating are characteristic of ESD with composite electrodes and can be explained by the presence of the lower-hard plasticizing TiAl phase, the hard boride, carbide and nitride phases, the change in the initial chemical composition, the partial combustion of boron in the transfer process, the oxidation and dissociation of TiB_2_ and the formation of new phases with different hardness, as well as the presence of pores and inhomogeneity in the structure of the applied coatings and the influence of the base of soft titanium alloy on hardness. The uneven distribution of the individual phases and elements in the layer also has a significant impact on the measured values. The comparison of the distribution of the elements in Zones 1 and 2—Figure 8, shows that in Zone 2 the amount of boron and aluminum is about two times higher than in Zone 1 (Figure 8a), and the amount of Ti is lower than in Zone 1, which suggests a higher microhardness in Zone 2. From the element distribution maps (Figure 9 and Figure 11) it can be seen that the most uneven is the distribution of Al and Ti, as in the zones with a predominant Al, titanium is less (Figure 9b,c and Figure 11c,d) and vice versa; where Ti predominates, aluminum is in a lower concentration. Boron, carbon and nitrogen are distributed more evenly, but their distribution mainly repeats that of Ti, which suggests the presence of titanium carbides, nitrides and carbonitrides and, accordingly, higher values of microhardness in these zones. Despite the tendency of titanium to oxidize, the oxygen distribution repeats the distribution of both titanium and Al (Figure 9c,e and Figure 11d,g). Probably, the presence of Al_2_O_3_ registered by XRD is precisely in these zones and suggests increased hardness. As can be seen, the uneven distribution is a major factor that does not allow to establish a clear dependence and correct assessment of the influence of the pulse energy.

However, the higher content of the high-hard phases also determines the higher obtained hardness values, i.e., the observed trend is towards an increase in microhardness with increasing pulse energy, but a clear and unambiguous dependence and a correct assessment which does allow to establish of the influence of the pulse energy on microhardness on the pulse energy is not established.

An approximate comparison of the influence of the pulse energy and pulse parameters shows that the highest hardness HV and HU are demonstrated by the coatings deposited in mode 4 (Sample 4) followed by those in mode 1 (Sample 1)—Table 1, whose composition is richest in high-hardness phases. The higher average and maximum values of HV and HU in Sample 4 are obviously due to the better cohesive strength, the greater amount of amorphous–nanocrystalline structures and the relatively lower porosity of these coatings. The lowest values of HV and HU are observed in LESD with mode 5 (Sample 5), probably due to the greater amount of structural defects in this mode. In general, the differences in the average values of HV and HU of the studied samples are in a relatively narrow range up to ≈2.5 GPa. Moreover, the values of HV are slightly higher than those of the universal hardness HU, but the ratios between them are proportional. The metal bond as part of the coating material reduces the hardness of the coatings but can provide high adhesion to the substrate and serves as a matrix that firmly holds the particles of borides, carbides and nitrides and prevents their chipping off under load. A large scatter in the values of HV and HU is reported by almost all researchers. Our results are similar to those in most studies [17,18,19] as well as [25,26,28], where the microhardness of coatings from different electrode materials is most often reported at 8–12 GPa. The measured microhardness values in these works slightly exceed those obtained with conventional hard electrodes WC-Co8—Figure 12—and those based on TiB_2_ obtained in the works [32,33,34], but the differences are relatively small and reach only to 1–2 GPa.

The data obtained from these studies allow to determine the range of pulse energy and pulse parameters, in which the amount of high-hardness compounds of the electrode material and the formed new carbides, borides, intermetallic compounds and fine-grained ultradisperse and probably amorphous–crystalline structures, as well as the geometric characteristics of the coatings, are combined in a compromising way. In the specified range, the most favorable from the point of view of the composition, structure and thickness of the coatings is mode 4 (Sample 4), and in terms of the roughness parameters and structural defects of the coatings—mode 3 (Sample 3). The summary of the results obtained shows that the use of a TiB_2_-TiAl electrode and contactless electrospark deposition LESD with pulse energy up to 0.025 J allows the creation of coatings from a predominantly liquid phase with better uniformity, lower roughness and porosity and higher microhardness compared to coatings obtained with conventional hard alloys and electrodes known in the literature.

## 4. Conclusions

- By LESD with ultradisperse TiB_2_-TiAl electrodes and using low-energy pulses in the range I = 11.2–16 A, C = 0.5–2.2 µF, Ti = 8–12 µs, dense and uniform coatings can be obtained with reduced roughness, minimal structural defects and with thickness, roughness and microhardness that can be controlled through the energy parameters of the LESD mode in the ranges δ = 9–19.5 µm, Ra = 1.8–3.2 µm and HV = 9–13 GPa, respectively.
- The results show that at a relatively low capacitance of up to 2.2 µF and pulses of 12 µs, a continuous and uniform layer with fewer defects and cracks with a thickness of up to 20 µm can be obtained.
- The micro and nanohardness of the coatings is 3–4.5 times higher than that of the titanium base, with the differences in the values of the coatings deposited in the used modes varying in the range of 1.5–2.5 GPa. The highest microhardness is in LESD with a pulse energy of 0.025 J and pulse parameters I = 16 A, C = 2.2 µF, Ti = 12 µs.
- During the LESD process, a large number of newly formed wear-resistant phases and intermetallic compounds are synthesized in the coatings, as well as an increased amount of amorphous–nanocrystalline structures and improved micro and nanohardness. The largest amount of amorphous–nanocrystalline regions is registered in the coatings in the mode with pulse energy E = 0.025 J and current values I = 16 A, capacitance C = 2.2 µF, pulse duration Ti = 12 µs and pulse frequency f = 8.33 kHz.
- Based on the results of experimental studies, it can be stated that by using TiB_2_-TiAl electrodes by the LESD method with low pulse energy, it was achieved to obtain coatings with reduced surface defects, with improved topography, morphology, composition and structure, increased hardness as a result of obtaining new compounds and amorphous–crystalline structures and significantly improved quality of titanium surfaces. The results of the present study confirm the positive effect of coatings from TiB_2_-TiAl electrodes on the geometric characteristics, microhardness, composition and structure of coated titanium surfaces.
- The obtained results create opportunities for preliminary selection of LESD modes to obtain coatings with the desired thickness, composition and roughness structure, to optimize the quality of the surface in order to create prerequisites for obtaining maximum triboeffect and increasing the operational properties corresponding to the type and requirements for the coated titanium surface.
- In order to evaluate the effect of the improved characteristics of the coatings on their tribological and corrosion properties, it is necessary to conduct additional studies.

## Figures and Tables

**Table 1:** Parameters of the selected modes for LESD.

N	Designation	Current, I, A	Capacitance, C, μF	Pulse Duration, Ti, μs	Frequency, f, kHz	Pulse Energy, E, J
**1**	Sample 1	16	4.4	12	8.33	0.025
**2**	Sample 2	11.2	0.5	12	8.33	0.013
**3**	Sample 3	12.8	0.5	8	12.5	0.013
**4**	Sample 4	16	2.2	12	8.33	0.02
**5**	Sample 5	12.8	4.4	8	12.5	0.02

**Table 2:** Phase composition of the coatings—Samples 2, 3 and 4.

Main Phases	2θ°	Phases in Small Amounts	2θ°	Traces of Phases	2θ°
**α-Ti**	35.38; 38.5; 40.2; 53; 63; 70.8; 74; 76.2; 77.2	AlTi	38.72; 45.6; 65.35; 66.05; 70.5; 78.45; 79.20	Al	38.5; 44.7; 65
**AlTi_3_**	26.33; 31.12; 35.65; 38.8; 39.4; 40.8; 43.05; 53.8	Ti_6_O	34.9; 37; 38.4; 40; 52.5; 70.1; 75.6	TiC_0.7_N_0.3_	35; 42; 61; 72.9
**TiN**	36.95; 42.95; 62.2; 74.5; 78.5	Ti_3_O	37.8; 39.9; 52.1; 62.5; 69.6; 75.3	AlN	20.5; 33.2; 36.2; 38.1; 59.4; 61; 66; 70; 71.8; 72.7
**TiB_2_**	27.7; 33.38; 34.2; 44.6; 56.9; 61.1; 67.9; 72; 78.6	TiC_0.3_N_0.7_	36.5; 74.5	AlB	21.5; 23.3; 36.8
**TiN_0.3_**	35; 37.5; 39.5; 52.2; 62.5; 69.2; 75.5; 77	Al_2_O_3_	19.5; 32.1; 35.65; 37.6; 39.2; 43.05; 44.5; 45.6; 50; 56.7; 60.5; 66.8; 71.4; 75.3; 78.55	BN	43.1; 74; 76
**TiB**	37.05; 42.95; 62.25; 78.5	Ti_2_O	33.6,35.65; 38.45; 40.7; 53; 63.7; 70.5; 76.6; 77.15; 78.5	Al_2.86_O_3.45_N_0.55_	32; 37.5; 45.8; 66.5; 69.5
**Ti_3.2_B_1.6_** **N_2.4_** **(Ti_4_N_3_B_2_)_0.8_**	33.36; 37; 42.95; 62.25; 74.5; 78.5	TiC_1−x_	35.9; 41.7; 60.3; 72.2	Al_3_Ti	25.5; 33.5; 39.5; 42.2; 46; 48; 55; 65.8; 66.6; 70; 75.3

**Table 3:** Range of crystallite sizes of the phases in the LESD coatings of TiB_2_-TiAl electrodes deposited under the regimes from Table 1.

Phases	α-Ti	TiN	TiN_0.3_	TiC_0.3_N_0.7_	TiB_2_	TiB	Ti_3.2_B_1.6_N_2.4_	Al_3_Ti	Ti_3_O	Ti_2_O	Al_2_O_3_	TiC_1−x_	AlN	AlTi
**Crystallite size, nm**	26–87	13–66	31–46	6–51	15–78	14–55	31–53	26–49	17–44	33–40	22–76	10–44	14–37	22–49

## Data Availability

The original contributions presented in this study are included in the article. Further inquiries can be directed to the corresponding authors.
